# Does Acupuncture Benefit Delayed-Onset Muscle Soreness After Strenuous Exercise? A Systematic Review and Meta-Analysis

**DOI:** 10.3389/fphys.2020.00666

**Published:** 2020-07-17

**Authors:** Chunwei Huang, Zhipeng Wang, Xiaoling Xu, Shuangshuang Hu, Rong Zhu, Xi Chen

**Affiliations:** ^1^Department of Gastroenterology, Wenzhou Central Hospital, Wenzhou, China; ^2^School of Sports Science, Wenzhou Medical University, Wenzhou, China; ^3^Sports Hospital Affiliated With Zhejiang College of Sports, Zhejiang College of Sports, Hangzhou, China

**Keywords:** acupuncture, exercise, delayed-onset muscle soreness (DOMS), systematic review, meta-analysis

## Abstract

**Purpose:** This systematic review and meta-analysis was designed to evaluate the effects of acupuncture intervention on alleviating delayed onset of muscle soreness (DOMS) after intense exercise.

**Method:** Randomized controlled trials (RCTs) were searched from online databases including Medline (PubMed), Cochrane Library, Web of Science, Embase, PsycINFO, China Knowledge Resource Integrated Database (CNKI), and Wanfang (Chinese) up to April 2019. Data points were extracted from the eligible RCTs at the time points of 24, 48, and 72 h post strenuous exercise-induced DOMS. The outcomes of muscle soreness rating (MSR), creatine kinase (CK), and maximal isometric force (MIF) were pooled into the meta-analysis to assess the acupuncture intervention on DOMS.

**Results:** Six eligible RCTs were included in the meta-analysis, and the results showed that acupuncture intervention significantly decreased MSR [standardized mean difference (SMD) −0.49, 95%CI −0.73 to −0.24, *P* < 0.001, *I*^2^ = 34%] and the serum level of CK (SMD −0.91, 95%CI −1.27 to −0.56, *P* < 0.001, *I*^2^ = 30%), accompanied with the improvement of the muscle strength (MIF) (SMD 0.54, 95%CI 0.16 to 0.93, *P* = 0.006, *I*^2^ = 51%) after intense exercise. At the same time, the findings also revealed that acupuncture intervention had a long-lasting effect and tended to accumulate the effect size and that it had the most efficacy on alleviating DOMS at the time point of 72 h post exercise.

**Conclusion:** The current evidence indicates that acupuncture intervention after intense exercise could be effective for alleviating DOMS and improving muscle recovery. The long-lasting effect of acupuncture intervention on DOMS started from 24 h and would reach a peak on the time point of 72 h post exercise.

## Introduction

It is well-established that delayed-onset muscle soreness (DOMS) will occur in both amateur and professional athletes who experience a high intensity of exercise (Lewis et al., 2012). DOMS is mainly caused by exhaustive or unaccustomed muscle work, especially of eccentric contractions (Bleakley et al., 2012; Lewis et al., 2012).

DOMS commonly occurs within the initial 24 h after strenuous or extreme exercises and reaches a peak between 24 and 72 h (Howatson and van Someren, 2008; Lewis et al., 2012), and it is frequently associated with muscle swelling and weakness and loss of motion range, leading to negative interference with daily activities and athletic performance or even increased risk of injuries (Cheung et al., 2003; Lavender and Nosaka, 2006; Kargarfard et al., 2016; De Marchi et al., 2017). Studies showed that the primary mechanical damage of muscle fibers during strenuous eccentric contractions or repetitive muscle work, accompanied with subsequent inflammation, caused DOMS (Proske and Morgan, 2001; Yu et al., 2002; Peake et al., 2005; Kanda et al., 2013; Kawamura et al., 2018). Zimmermann et al. (2012) found that exercise-induced DOMS activated widespread brain areas by fMRI, which also produces pain experience.

Numerous physiotherapeutic interventions such as cold-water immersion, massage, vibration, stretching, acupuncture, and electrotherapy were used to avoid or alleviate DOMS (Weerapong et al., 2005; Howatson et al., 2009; Bleakley et al., 2012; Costello et al., 2012; Hill et al., 2014; Jeon et al., 2015; Guo et al., 2017). As one of the traditional Chinese medicines, acupuncture has been proven effective in various musculoskeletal disorders including muscle soreness; thus, acupuncture therapy has been widely performed to improve the recovery of muscle-related injury and DOMS (Hubscher et al., 2008; Wand et al., 2013; Law et al., 2015). In traditional Chinese medicine theory, needling or acupuncture could activate the meridian and create a “de-qi” sensation, which accelerated the recovery of the symptoms of exercise-induced DOMS (Chang et al., 2019). In addition, the impact of acupuncture on extra segmental and central neuromodulation through local axon reflexes also explained the curative effect of acupuncture in Western countries (Chon and Lee, 2013). However, based on the up-to-date evidence, there is no consensus about acupuncture treatment on relieving DOMS and improving muscle recovery after intense exercise. Hubscher et al. (2008) discovered that needle acupuncture reduced muscle pain and increased muscle strength after exercise-induced DOMS, whereas other studies showed that acupuncture had no significant effect on improving the symptoms of DOMS (Barlas et al., 2000; Fleckenstein et al., 2016). Different types of acupuncture, such as using a laser or not, and different acupuncture points may result in inconsistent outcomes (Itoh et al., 2011; Fleckenstein et al., 2016).

O'Connor and Hurley (2003) systematic review investigated the effectiveness of many physiotherapy interventions (including acupuncture) on DOMS and found no evidence to support that acupuncture helped reduce DOMS, but there were only two trials included in the review. Recently, another systematic review based on more randomized controlled trial (RCTs) (which were not pooled into a meta-analysis) indicated that the therapeutic effect of acupuncture analgesia in DOMS is conflicting (Ko and Clarkson, 2019). Therefore, this systematic review and meta-analysis aimed to update the current evidence to evaluate the effects of acupuncture intervention on reducing DOMS and improving muscle performance after strenuous exercise.

## Methods

The current systematic review and meta-analysis was conducted according to the Cochrane guidelines (Higgins et al., 2019).

### Search Strategy

An electronic literature search for the relevant research articles which were published from January 1980 to May 2019 with no language restriction was conducted on the following online databases: Medline (PubMed), Cochrane Library, Web of Science, Embase, PsycINFO, China Knowledge Resource Integrated Database (CNKI), and Wanfang (Chinese). The keywords for searching are acupuncture, exercise, muscle pain, DOMS, and RCTs, and the details of the search strategy were provided in the Supplementary Data (S1).

### Inclusion and Exclusion Criteria

Trials were included according to the following criteria: (a) only the studies were designed as RCTs; (b) participants should be healthy regardless of nationality; (c) the acupuncture intervention should be used after exercise for the recovery of the symptoms of DOMS; (d) the acupuncture intervention should be compared to control intervention (no intervention or placebo) regardless of any types of acupuncture; and (e) outcomes should cover muscle pain or soreness rating or maximal isometric force (MIF) or the serum creatine kinase (CK) level.

Trials were excluded according to the following criteria: (a) the data of mean and SD are inaccessible from the articles or the corresponding authors, and (b) the acupuncture intervention was used before exercise.

### Trial Selection

The titles and abstracts of all the selected articles were reviewed by two reviewers (Huang CH and Wang ZP) independently for an initial scan, and then the full contents of the articles were carefully checked to assess whether the articles satisfied the inclusion criteria or not. In case of disagreement with trial selection, the two reviewers discussed with each other or consulted another reviewer (Chen X).

### Quality Assessment

According to the Cochrane Collaboration recommendation (Higgins et al., 2019), all the included RCTs should be assessed with seven main risks of bias, including random sequence generation (selection bias), allocation concealment (selection bias), blinding of participants and personnel (performance bias), blinding of outcome assessment (detection bias), incomplete outcome data (attrition bias), selective reporting (reporting bias), and other biases. Two reviewers (Huang CH and Wang ZP) independently evaluated all the selected trials with three grades of biases (high or low or unclear), which were labeled by the Cochrane Collaboration tool (*Revman Version 5.3, The Nordic Cochrane Center, The Cochrane Collaboration, Copenhagen, 2014*). Any disagreement was settled by discussion or a consultation with another reviewer (Chen X).

Then the GRADE scores of the pooled meta-analyses were evaluated according to the Cochrane guidelines (Higgins et al., 2019).

### Data Extraction

All the data points were extracted by two reviewers (Huang CH and Wang ZP) independently from the eligible included trials, including study characteristics (the first author and publication year), participant characteristics (age and gender, groups, and number of participants), intervention descriptions, outcomes, and measurement time points. Then we extracted every trail's mean and SD for meta-analysis.

### Statistical Analysis

Review Manager software (*Revman Version 5.3, The Nordic Cochrane Center, The Cochrane Collaboration, Copenhagen, 2014*) was used to perform the meta-analysis. Standardized mean difference (SMD) was used to analyze the data points as the trials used different exercises to induce different muscle DOMS models and the scales for muscle soreness also vary between trials; *P* < 0.05 was considered to be a significant difference. The *I*^2^ statistic was used to evaluate the heterogeneity of the effects, and the *I*^2^ < 25, < 75, and > 25%, and ≥75% were considered as low, moderate, and high heterogeneity, respectively (Higgins et al., 2003). Meta-analysis was conducted when there are two or more outcomes from the trials by the random-effects model. Subgroups were performed to analyze the effectiveness of acupuncture on DOMS at different time points of 24, 48, and 72 h post strenuous exercise.

As the included articles are <9, funnel plot asymmetry was not used to evaluate the publication bias. Sensitivity analysis was used to assess the stability of results by removing each data point one by one. If the outcome showed the data of the mean and standard error (SEM), the standard deviation (SD) would be calculated as SD = SE × $\sqrt{n}$ (*n* = sample size).

## Results

### Search Results

**Figure 1:**
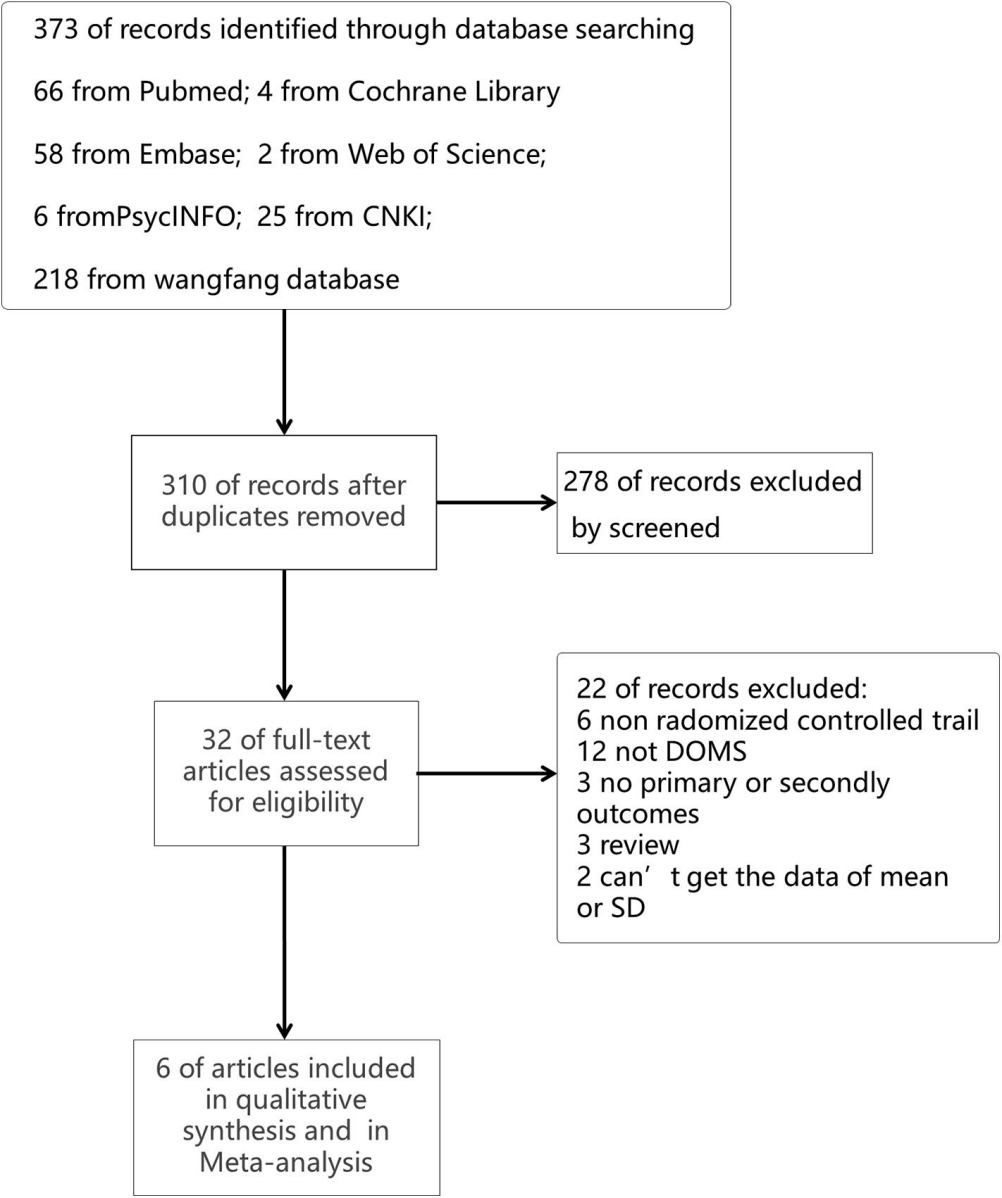
Flow diagram of the study selection.

A total of 373 searching records were obtained from our initial online database search, and 32 potentially eligible trials were identified by reviewing the information of articles' titles and abstracts. After carefully reviewing the contents of all the 32 articles, six articles with 210 participants finally satisfied the inclusion criteria and were included in this meta-analysis. The process of identifying these trials from searching on databases is illustrated in Figure 1.

### Description of Included Studies

Six articles published from 1999 to 2016 were included in this meta-analysis (Lin and Yang, 1999; Barlas et al., 2000; Wang et al., 2006; Itoh et al., 2008; Tang, 2009; Fleckenstein et al., 2016), and the characteristics were shown in Table 1. Among these studies, four articles were published in English and two in Chinese. The publication countries were China (*n* = 3, 50%), Germany (*n* = 1, 16.7%), Japan (*n* = 1, 16.7%), and United Kingdom (*n* = 1, 16.7%). All participants included were healthy, aged from 18 to 40 years old with 151 males and 59 females.

**Table 1:** Characteristic of included studies.

**NO**.	**Reference**	**Country/region**	**Patients characteristic, sample size**	**Induce DOMS**	**Intervention**	**Duration of intervention period**	**Outcomes**	**Time point**
1	Lin and Yang (1999)	Taiwan (China)	Twenty healthy male volunteers (ages 19–30) (Group A = 10,Group B = 10)	Performed strenuous eccentric exercise of nodominant elbow flexion	Group A: no intervention; Group B: acupuncture treatment	20 min	Mucle soreness rating (VAS), creaine kinase level (CK)	24 h, 48 h,72 h after DOMS induced exercise
2	Fleckenstein et al. (2016)	Germany	Sixty healthy participants (22 females and 38 males, age 23.6 ± 2.8 years) (G1 = 12, G2 = 12, G3 = 12, G4 = 12,G5 = 12)	Performed isolated biceps curls with a dumbbell of non-dominant elbowflexors	G1:verum acupuncture, G2: sham acupuncture, G3:laser acupuncture, G4:sham laser acupuncture, G5:no intervention	20 min	Mucle soreness rating (VAS, PPT), Maximum isometric voluntary force (MIF)	0 h, 24 h, 48 h,72 h after DOMS induced exercise
3	Itoh et al. (2008)	Japan	Thirty participants (aged 18–22 years with 13 female, 17 male) (G1 = 10, G2 = 10, G3 = 10)	Performed eccentric exercise of nodominant elbow flexion	G1: no intervention; G2: non-tender point acupuncture; G3:tender point group acupuncture	10 min	Mucle soreness rating (VAS)	0 h, 24 h, 48 h, 72 h, and 7 days after DOMS induced exercise
4	Barlas et al. (2000)	British	Volunteers students and staff (aged 18–40 years, with 24 female, 24 male) (G1 = 12, G2 = 12,G3 = 12,G4 = 12)	Nodominant arm using dumb-bell and free weights	G1: no intervention; G2: placebo group (needling at non-acupuncture points); G3: treatment group 1(needling at traditional acupuncture points); G4: treatment group 2(needing at ‘tender’ points)	20 min	Mucle soreness rating (VAS, PPT), Range of movement (ROM)	0 h, 24 h, 48 h, 72 h, 96 h, and 5 days after DOMS induced exercise
5	Wang et al. (2006)	China	Thirty healthy male students (19–23 years) (G1 =10, G2 = 10, G3 = 10)	Five sets of frog-jumps (totally 60 jumps), treadmill running along 15° slope at the speed of 10 km/h until exhaust	G1:PNF stretch exercise, G2: acupuncture group: oblique needling in the painful point of the muscle; G3: control group	15 min	Mucle soreness rating (PPT), creaine kinase level (CK), high jump	0 h, 24 h, 48 h, 72 h after DOMS induced exercise
6	Tang (2009)	China	Twenty-two healthy male students (18–25 years) (G1 = 8, G2 = 7, G3 = 7)	Seven sets of 50m frog-jumps with 2–3 min interval	G1 on intervention; G2: acupuncture group 1: needling one point (only zusanli); G3: acupuncture group 2: needing four points (including zusanli).	20 min	Mucle soreness rating (VAS), creaine kinase level (CK), Maximum isometric voluntary force (MIF)	0 h, 2 h, 24 h, 48 h, 72 h, 96 h after DOMS induced exercise

### Acupuncture and Control Intervention

All trials use the acupuncture intervention and needling acupuncture point (tender point or Ashi point), and one trial also added a third group using laser acupuncture. The duration of needles being retained in place ranged from 10 to 20 min. Half (three) trials twirled needles to manually provoke “de-qi” stimulation every 5 min.

All trials assigned no intervention (just sit for rest) for the control group. One trial added laser sham acupuncture group as the control for laser acupuncture.

### Risk of Bias of Included Trials

**Figure 2:**
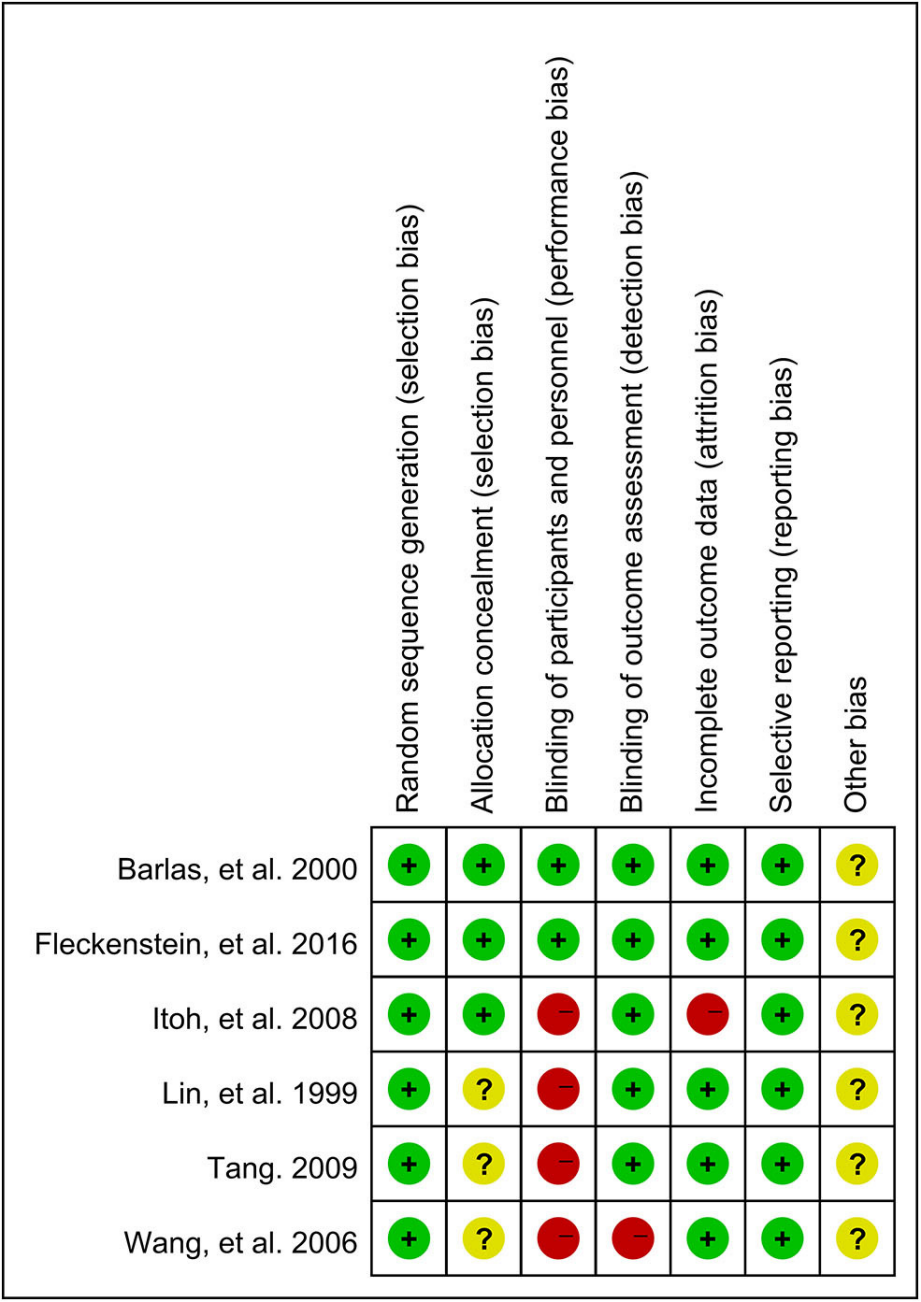
The risk of bias summary of included trials.

The risk of bias of the included trials was evaluated according to the recommendation by Higgins et al. (2011). All of the six articles used the randomization method, but only three of them reported the information about allocation concealment (50%). Only two studies (33.3%) used the blind method for participants as they set a group of placebo or sham acupuncture, which increased the risk of performance bias. Most of the trials (86.7%) masked their outcome assessors and showed the low risk of detection bias, as well as the low risk of attrition (86.7%) and reporting bias (100%). As funnel plot asymmetry was not applicable, publication bias was unclear. It was unclear whether the trials had other biases. The detail of the assessment of all the risks of bias was shown in Figure 2.

### Effect of Acupuncture Intervention on Muscle Soreness Rating

**Figure 3:**
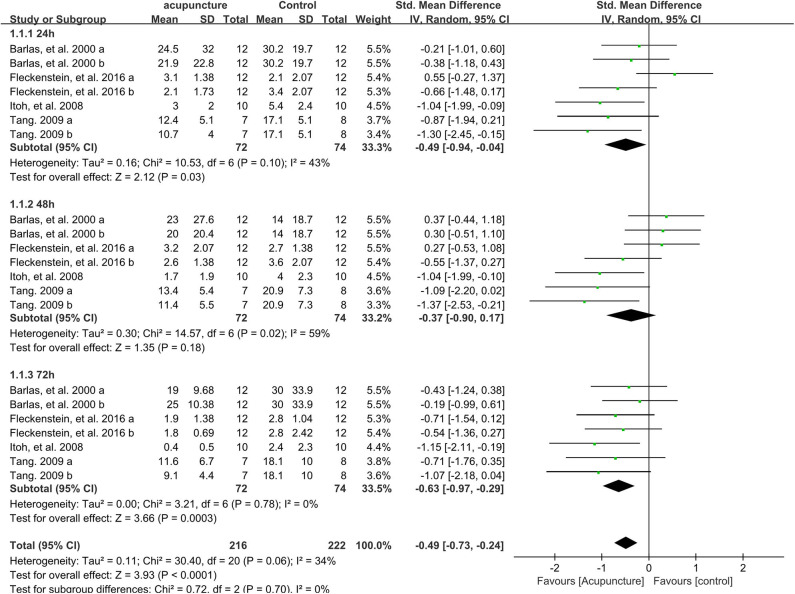
Meta-analysis of the effect of acupuncture intervention on MSR (divided three subgroups and the total effect). MSR, muscle soreness rating.

As shown in Figure 3, 21 data points demonstrated that acupuncture intervention significantly alleviated muscle soreness rating (MSR) when compared with the control group (SMD −0.49, 95%CI −0.73 to −0.24, *P* < 0.001, *I*^2^ = 34%). Among these, the subgroups of 24 h (SMD −0.49, 95%CI −0.94 to −0.04, *P* = 0.03, *I*^2^ = 43%) and 72 h (SMD −0.63, 95%CI −0.97 to −0.29, *P* < 0.001, *I*^2^ = 0%) time points also showed that compared to that in the control group, MSR significantly decreased when the participants received acupuncture intervention. The subgroup of the 48 h time point illustrated no significant difference between acupuncture intervention and no intervention although acupuncture intervention showed a slight reduction trend of MSR (SMD −0.37, 95%CI −0.90 to 0.17, *P* = 0.18, *I*^2^ = 59%).

Sensitivity analysis of the MSR outcome revealed that the effect was stable when data points were removed one by one.

### Effect of Acupuncture Intervention on the Pressure Pain Threshold

**Figure 4:**
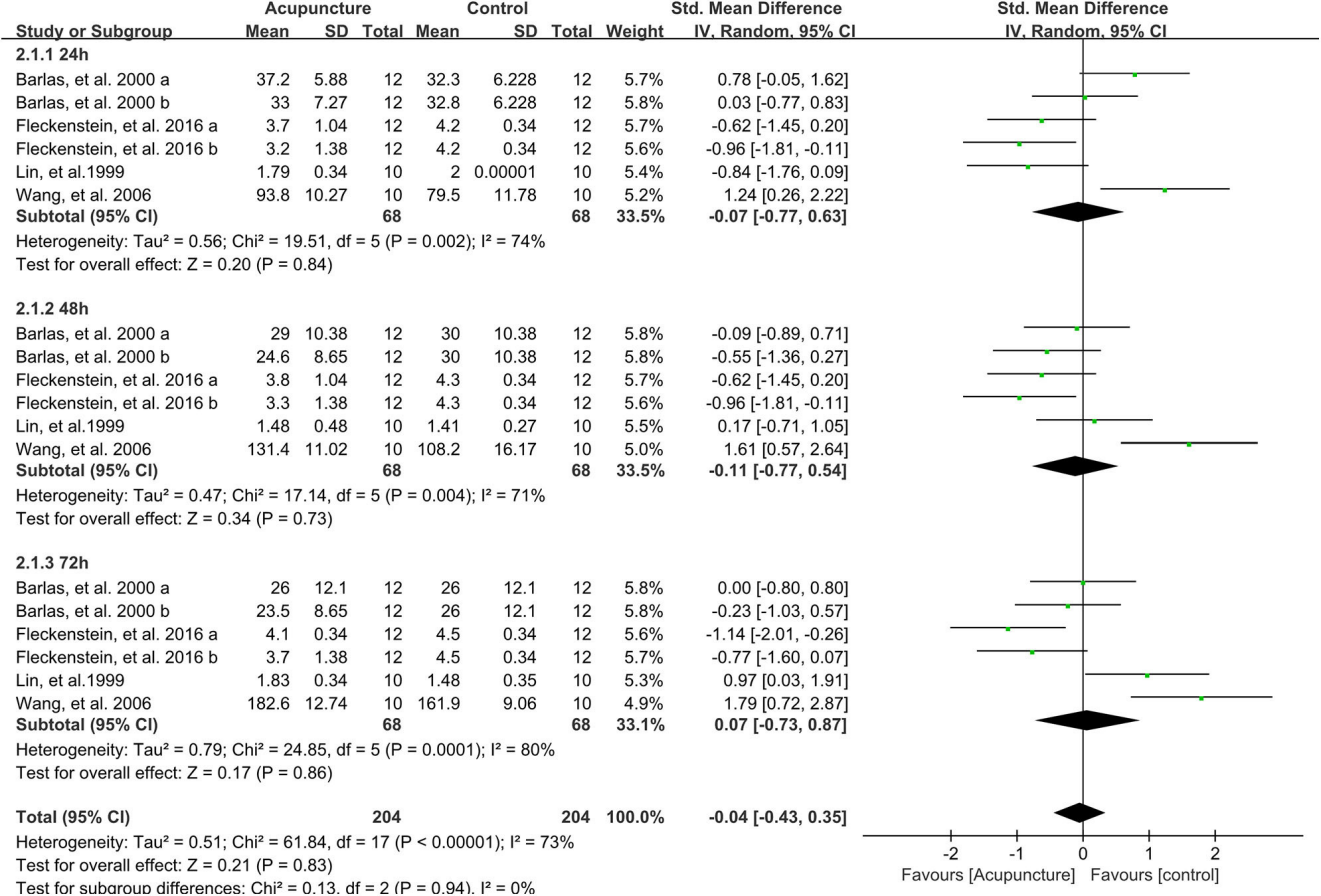
Meta-analysis of the effect of acupuncture intervention on PPT (divided into three subgroups and the total effect). PPT, pressure pain threshold.

Eighteen data points compared the pressure pain threshold (PPT) between acupuncture and control groups after intense exercise; no significant difference was found in terms of the total effect based on the meta-analysis (SMD 0.08, 95%CI −0.27 to 0.43, *P* = 0.42, *I*^2^ = 66%) (Figure 4). Besides, all subgroups showed no significant difference on PPT after strenuous exercise whether the participants received the acupuncture intervention or not on the time points of 24 h (SMD 0.05, 95%CI −0.57 to 0.66, *P* = 0.88, *I*^2^ = 67%), 48 h (SMD −0.01, 95%CI −0.59 to 0.57, *P* = 0.97, *I*^2^ = 63%) or 72 h (SMD 0.21, 95%CI −0.53 to 0.94, *P* = 0.58, *I*^2^ = 76%) post exercise (Figure 4).

Sensitivity analysis demonstrated that the results were stable when the data points were removed one by one.

### Effect of Acupuncture Intervention on Serum Level of CK

**Figure 5:**
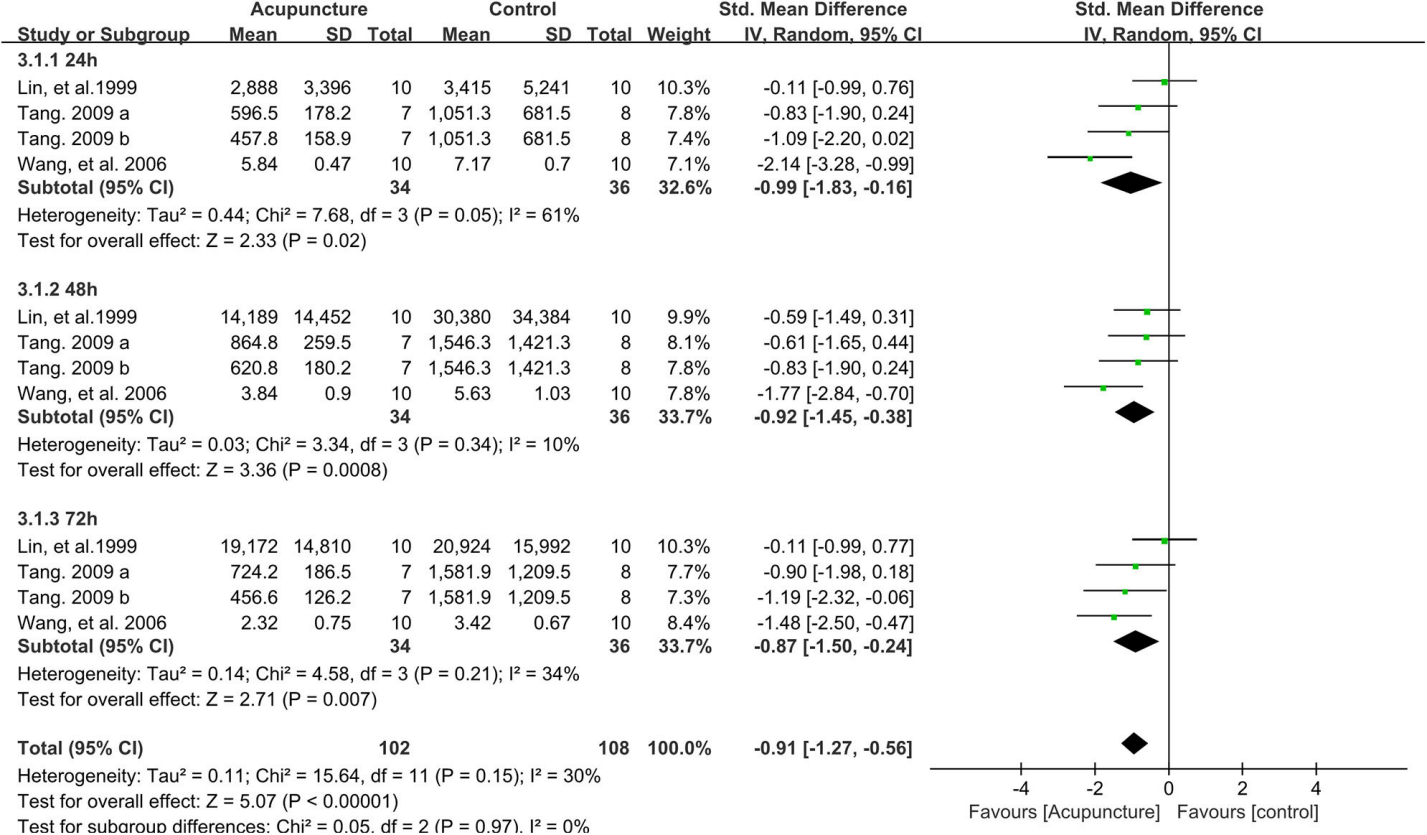
Meta-analysis of the effect of acupuncture intervention on the serum CK level (divided into three subgroups and the total effect). CK, creatine kinase.

As shown in Figure 5, four data points compared the effect of acupuncture intervention on the serum level of CK of the control group on the time points of 24, 48, and 72 h post strenuous exercise. The subgroup meta-analysis demonstrated that acupuncture intervention significantly decreased the serum level of CK on the time points of 24 h (SMD −0.99, 95%CI −1.83 to −0.16, *P* < 0.02, *I*^2^ = 61%), 48 h (SMD −0.92, 95%CI −1.45 to −0.38, *P* < 0.001, *I*^2^ = 10%), and 72 h (SMD −0.87, 95%CI −1.50 to −0.24, *P* < 0.007, *I*^2^ = 34%) after strenuous exercise. Consistent with the subgroup meta-analysis, the total effect of the 12 data points also revealed that acupuncture intervention could significantly decrease the serum level of CK after strenuous exercise (SMD −0.91, 95%CI −1.27 to −0.56, *P* < 0.001, *I*^2^ = 30%).

Sensitivity analysis showed that the effect of acupuncture intervention on the serum level of CK was stable when the data points were removed one by one.

### Effect of Acupuncture Intervention on the MIF

**Figure 6:**
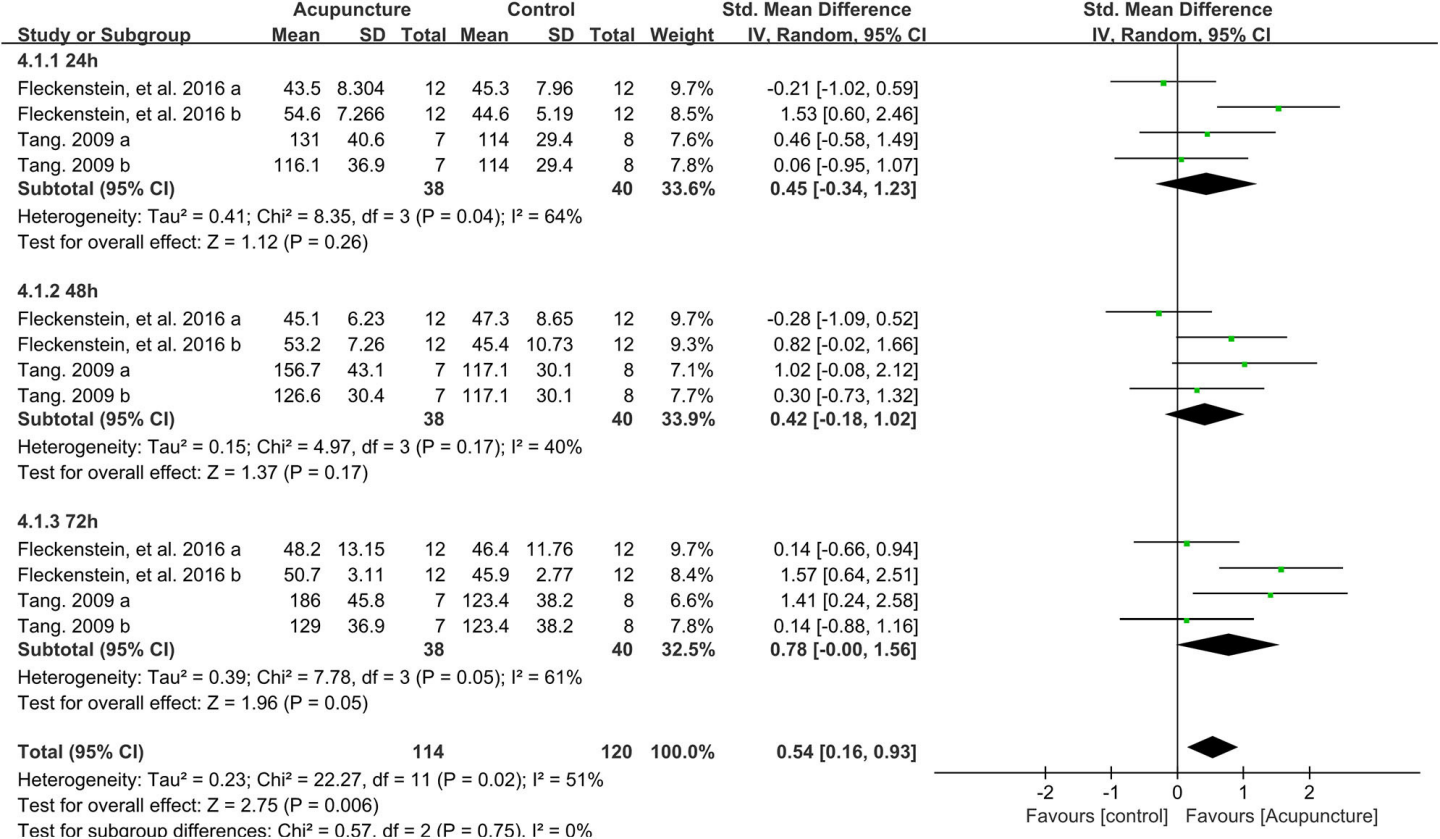
Meta-analysis of the effect of acupuncture intervention on MIF (divided into three subgroups and the total effect). MIF, maximal isometric force.

Four data points compared the performance of MIF between acupuncture and control groups on the time points of 24, 48, and 72 h after strenuous exercise. Based on subgroup meta-analysis, it was found that acupuncture intervention significantly improved the MIF performance only on the time point of 72 h (SMD 0.78, 95%CI −0.00 to 1.56, *P* = 0.05, *I*^2^ = 61%) post exercise, with a slight increase of MIF but no significant difference observed on the time points of 24 h (SMD 0.45, 95%CI −0.34 to 1.23, *P* = 0.26, *I*^2^ = 64%) or 48 h (SMD 0.42, 95%CI −0.18 to 1.02, *P* = 0.17, *I*^2^ = 40%) (Figure 6). However, the total effect of the 12 data points showed that acupuncture intervention significantly enhanced the MIF performance when compared to the control group (SMD 0.54, 95%CI 0.16 to 0.93, *P* = 0.006, *I*^2^ = 51%) (Figure 6).

Sensitivity analysis revealed that the result of the total effect of acupuncture intervention on MIF was stable when the data points were removed one by one.

### Result of GRADE Scores

The result of GRADE scores displayed that the qualities of evidence in the four outcomes of meta-analyses were low (Table 2). Inconsistency and imprecision were the main factors contributing to the low quality of evidence.

**Table 2:** The GRADE scores of the outcomes of MSR, PPT, serum level of CK, and MIF.

**Intervention:** Acupuncture			
**Outcomes**	**Absolute effects with comparative risks** **(95% CI)**	**No. of participants** **(data points)**	**Quality of the evidence** **(GRADE)^a^^,^^b^**
Muscle soreness rate (MSR)	The mean muscle soreness rate in the acupuncture intervention groups was **0.49** (95% CI 0.73 to 0.24 lower)	438 (21 data points)	(–)^*a*^(–)^*b*^ LOW quality of the evidence due to inconsistency and imprecision
Pressure pain threshold (PPT)	The mean muscle soreness rate in the acupuncture intervention groups was **0.08** (95% CI −0.27 to 0.43 lower)	408 (18 data points)	(–)^*a*^(–)^*b*^ LOW quality of the evidence due to inconsistency and imprecision
Serum level of CK	The mean muscle soreness rate in the acupuncture intervention groups was **0.91** (95% CI 1.27 to 0.56 lower)	210 (12 data points)	(–)^*a*^(–)^*b*^ LOW quality of the evidence due to inconsistency and imprecision
Maximal isometric force (MIF)	The mean muscle soreness rate in the acupuncture intervention groups was **0.54** (95% CI 0.16 to 0.93 lower)	234 (12 data points)	(–)^*a*^(–)^*b*^ LOW quality of the evidence due to inconsistency and imprecision

*GRADE Working Group grades of evidence*.

***High quality:** Further research is very unlikely to change our confidence in the estimate of effect. **Moderate quality:** Further research is likely to have an important impact on our confidence in the estimate of effect and may change the estimate. **Low quality:** Further research is very likely to have an important impact on our confidence in the estimate of effect and is likely to change the estimate. **Very low quality:** We are very uncertain about the estimate*.

*Downgrade quality of evidence was used for GRADE scores*.

a *Inconsistency risk (the relative effect of the acupuncture intervention and its 95%CI across the median control group)*.

b *Imprecision risk (SD > 10% effect size, SD < effect size)*.

### Adverse Events

All included trials did not state any adverse events.

## Discussion

Exercise-induced muscle damage and soreness occur in both amateurs and professional athletes after strenuous exercise; thus, many studies have been focusing on reducing muscle soreness and increasing the recovery of muscle strength for the next event (Jakeman et al., 2010). In traditional Chinese medicine, massage, cupping, and acupuncture have been widely used in treating muscle pain and improving muscle recovery after exercise (Chen et al., 2016). Systematic reviews revealed that massage and cupping could be beneficial to muscle soreness and the performance of muscle strength after intense exercise (Guo et al., 2017; Bridgett et al., 2018). Animal studies suggested that acupuncture intervention helped to recover the reduction of perimysial junctional plate (PJP) number and the alteration of PJP domain homeostasis induced by eccentric training, leading to an increase of the muscle damage repair (Kong et al., 2017). Moreover, laser acupuncture also showed a beneficial effect as it reduced inflammation and myofascial pain in rats (Lorenzini et al., 2010). Nevertheless, there is still a lack of evidence to support these mechanisms in the human study, and the corresponding guidelines for medical practitioners remain elusive.

To address the conflicting results across various studies regarding the effect of acupuncture intervention on DOMS (Barlas et al., 2000; Hubscher et al., 2008; Fleckenstein et al., 2016), we have conducted this systematic review and meta-analysis to better understand the effectiveness of acupuncture intervention on DOMS and muscle strength recovery after exercise. The key findings demonstrated that participants receiving acupuncture intervention had experienced reduced muscle soreness after strenuous exercise. In addition, the results of a subgroup meta-analysis revealed that the beneficial effect of acupuncture intervention on alleviating muscle pain had more efficacy on the time points of 72 h (SMD −0.63) than 24 h (SMD −0.49) and 48 h post exercise, indicating that the effect of acupuncture on muscle pain tends to be long-lasting and accumulated peak at 72 h post intense exercise, which is consistent with Law et al. (2015) systematic review showing that laser acupuncture had a long-term effect on musculoskeletal pain. However, the results of PPT in this study show no significant difference between acupuncture therapy and the control group, indicating that the mechanisms of acupuncture therapy may vary in alleviating muscle pain and increasing PPT. Furthermore, the different types of acupuncture intervention may affect the effect size of PPT, because of the high heterogeneity of the PPT outcome and because the positive effects only come from two Chinese trials on the time point of 72 h post exercise (Lin and Yang, 1999; Wang et al., 2006).

It is now clear that the serum level of CK will increase rapidly and that muscle strength decreases when DOMS occurs because of the damage in the skeletal muscle and the subsequent inflammation after intense exercise (Clarkson and Sayers, 1999; Romagnoli et al., 2015; Muller et al., 2019). The current evidence showed that acupuncture intervention reduced the serum level of CK based on the total effect meta-analysis, as well as the subgroup effect on the time points of 24, 48, and 72 h post exercise-induced DOMS, indicating that acupuncture intervention could improve the recovery of muscle damage. This could be explained by the findings of Kong et al. (2017) and Wen et al. (2019) studies, which showed that acupuncture intervention could improve the recovery from muscle damage and inflammation. We also found that acupuncture intervention increased MIF after strenuous exercise when compared to the control intervention. Improved muscle damage recovery was thought to be one of the reasons that caused increased MIF, the reduction of muscle soreness might be another reason, and the benefit of acupuncture intervention on the reduction in serum inflammation mediators such as interleukin 6 (IL-6) and interleukin 8 (IL-8) may also contribute to muscle performance recovery (Li et al., 2015; Mo et al., 2017). Furthermore, the subgroup meta-analysis of the 72 h post exercise time point showed a significant increase of MIF, with no significant differences observed on the time points of 24 and 48 h after exercise. This result is consistent with the muscle soreness rating and the serum level of CK we have observed before, strengthening the evidence that acupuncture has a long-lasting effect on DOMS; the effect size accumulated peaked on the time point of 72 h post intense exercise.

### Strengths and Limitations

The present study systematically reviewed the effect of acupuncture intervention on DOMS after strenuous exercise and pooled the RCTs into a meta-analysis, aiming to get a better understanding of the effect of acupuncture therapy on alleviating DOMS. Two reviewers searched and selected the trials independently, assessed the quality, extracted the data points, and pooled the trials into a meta-analysis according to the protocols and methodological schemes recommended, which strengthen the power of this meta-analysis. Different from Law et al. (2015) study, this study focused only on treating or preventing muscle pain induced by strenuous exercise (DOMS) but did not include other musculoskeletal disorders. The stability of the effects revealed by sensitivity analysis also strengthened the evidence that acupuncture intervention after exercise helps muscle recovery and alleviates DOMS. Moreover, based on the subgroup effect of different time points after intense exercise, this review discovered a long-lasting effect of acupuncture intervention on DOMS and revealed an accumulative effect.

However, there are some limitations in this present systematic review. First of all, the number of trials was relatively small; there are only eight articles that met the inclusion criteria, but we could not extract the mean and SD from two articles (Hubscher et al., 2008; Itoh et al., 2011) or by e-mailing the corresponding authors; thus, only six articles were pooled into the meta-analysis. More RCTs were needed for more evidence on assessing the effect of acupuncture on DOMS. Besides, the quality of the included articles is not high. Half trials did not detail any information on allocation concealment, and only two of them used sham acupuncture as a binding method for participants, which increased selection and performance bias. Selected trials contain varied acupuncture methods (dry needling or laser acupuncture) and acupuncture points (one point, four points, or just Ashi points), leading to the high heterogeneity. However, Chang et al. (2019) showed no significant difference between laser acupuncture and acupuncture intervention on DOMS, and the stability of total effects by sensitivity analyses also supports pooling the data points into a meta-analysis. Furthermore, the inconsistency and imprecision of the pooled meta-analyses also downgraded the quality of evidence in the results of the GRADE score. Finally, the publication bias could not be assessed by funnel plot asymmetry because the total included articles are <9.

## Conclusion

The current evidence suggests that acupuncture intervention alleviates DOMS and improves muscle recovery as well as performance after strenuous exercise. To date, no adverse events have been recorded. This meta-analysis also demonstrated that the long-lasting effect of acupuncture intervention on DOMS started from 24 h and would reach a peak on the time point of 72 h post exercise. These findings may support the potential use of acupuncture intervention for sports or exercise practice. Nonetheless, we should be cautious when using the conclusion because of the outlined limitations in this systematic review.

## Data Availability Statement

All datasets generated for this study are included in the article/Supplementary Material.

## Author Contributions

XC and RZ designed this systematic review and supervised the whole program. CH and ZW reviewed all the trials and extracted the information and the data points. SH searched the articles from all the online databases. XX analyzed the data and prepared the figures and the table. CH and XC wrote the paper. All the authors reviewed and approved the manuscript.

## Conflict of Interest

The authors declare that the research was conducted in the absence of any commercial or financial relationships that could be construed as a potential conflict of interest.
