# Supplementary material for: Does Acupuncture Benefit Delayed-Onset Muscle Soreness After Strenuous Exercise? A Systematic Review and Meta-Analysis
Source: Front Physiol. 2020 Jul 17;11:666. doi: 10.3389/fphys.2020.00666 (PMC7379881; doi:10.3389/fphys.2020.00666)
Supplement: Supplementary file 1 [file Data_Sheet_1.docx]

Does Acupuncture Benefit Delayed Onset Muscle Soreness After Strenuous Exercise? A Systematic Review and Meta-analysis

**Search strategies for all databases**

1. Search Strategy for pubmed:

#1 (((((muscle skeletal) OR athletic injuries) OR soft tissue injuries) OR Creatine kinase) OR muscle fatigue) OR muscle weakness (331573)

#2 ((damage) OR injury) OR injuries (1839972)

#3 muscle (1065495)

#4 #2 and #3 (124098)

#5 muscle soreness (9217)

#6 #1 or #4 or #5 (423078)

#7 **((((exercise) OR sports) OR training) OR competition) OR match (2164598)**

#8 #6 and #7 (92814)

#9 acupuncture (30220)

#10 #8 and #9 (183)

#11 randomized OR random OR randomly OR randomized controlled trial OR controlled clinical trial (1238868)

#12 #11 and #10 (84)

#13 (animal) NOT human (4348497)

#14 #12 NOT #13 (66)

2. Search Strategy for Cochrane library:

#1 injuries OR injury OR creatine kinase OR soreness OR damage (69676)

#2 fatigue or weakness (34015)

#3 #1 or #2 (2434)

#4 exercise AND muscle (18472)

#5 #3 AND #4 (542)

#6 acupuncture (13394)

#7 #5 AND #6（4）

3. Search Strategy for EMbase:

#1 exercise or sports (580770)

#2 injuries OR injury OR creatine kinase OR soreness OR damage or fatigue or weakness (971173)

#3 muscle (1358511)

#4 #1 AND #2 AND #3 (23381)

#5 random OR randomized OR randomization OR control OR controlled (8133970)

#6 acupuncture (41586)

#7 #4 and #5 and #6 (58)

4. Search Strategy for Web of science:

#1 exercise or sports (434661)

#2 injuries OR injury OR creatine kinase OR soreness OR damage or fatigue or weakness (1663832)

#3 muscle (779196)

#4 #1 AND #2 AND #3 (22234)

#5 random OR randomized OR randomization OR control OR controlled (5614875)

#6 acupuncture (15709)

#7 #4 and #5 and #6 (2)

5. Search Strategy for: psycINFO (6)

(exercise or sports) AND (injuries OR injury OR creatine kinase OR soreness OR damage or fatigue or weakness) AND (muscle) AND (random OR randomized OR randomization OR control OR controlled) AND (acupuncture)

6. Search Strategy for CNKI (25)

FT=随机+对照+随机对照+随机对照研究+随机对照试验+随机分配 AND SU=针灸治疗+针灸+针刺AND SU=延迟性肌肉酸痛+延迟性肌肉疼痛+运动后肌肉酸痛+运动后肌肉疼痛+DOMS+运动后肌肉损伤+延迟性肌肉损伤

7. Search Strategy for wanfang database (218)

主题：（随机+对照+随机对照+随机对照研究+随机对照试验+随机分配） AND 主题：（针灸治疗+针灸+针刺） AND 主题：（延迟性肌肉酸痛+延迟性肌肉疼痛+运动后肌肉酸痛+运动后肌肉疼痛+DOMS+运动后肌肉损伤+延迟性肌肉损伤）
